# tRNA/mRNA Mimicry by tmRNA and SmpB in *Trans*-Translation

**DOI:** 10.4061/2011/130581

**Published:** 2011-01-05

**Authors:** Daisuke Kurita, Akira Muto, Hyouta Himeno

**Affiliations:** Department of Biochemistry and Molecular Biology, Faculty of Agriculture and Life Science, Hirosaki University, Hirosaki 036-8561, Japan

## Abstract

Since accurate translation from mRNA to protein is critical to survival, cells have developed translational quality control systems. Bacterial ribosomes stalled on truncated mRNA are rescued by a system involving tmRNA and SmpB referred to as *trans*-translation. Here, we review current understanding of the mechanism of *trans*-translation. Based on results obtained by using directed hydroxyl radical probing, we propose a new type of molecular mimicry during *trans*-translation. Besides such chemical approaches, biochemical and cryo-EM studies have revealed the structural and functional aspects of multiple stages of *trans*-translation. These intensive works provide a basis for studying the dynamics of tmRNA/SmpB in the ribosome.

## 1. Introduction

**Figure 1:**
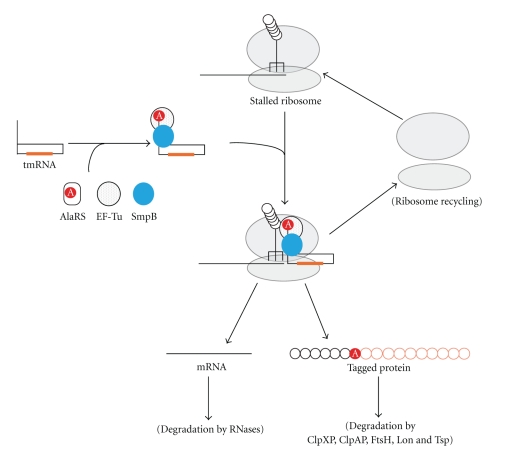
Schematic model of *trans*-translation. Ala-tmRNA receives the nascent polypeptide from peptidyl-tRNA in the P-site of the stalled ribosome to add a tag peptide.* Trans*-translation allows recycling of the ribosomes and promotes degradation of both truncated mRNAs and aberrant polypeptides from truncated mRNAs.

**Figure 2:**
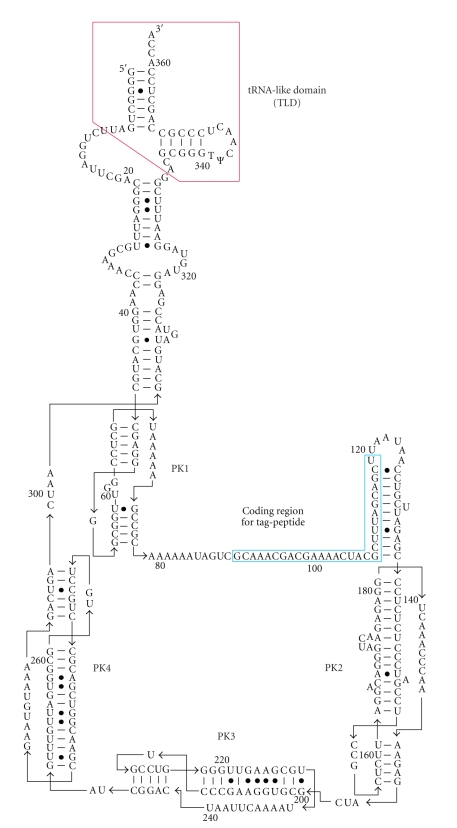
Secondary structure model of tmRNA from *E. coli*. The tRNA-like domain and mRNA domain are highlighted with red and blue, respectively. The tag-encoding sequence is surrounded by four pseudoknot structures (PK1-4).

Translation from the genetic information contained in mRNA to the amino acid sequence of a protein is performed on the ribosome, a large ribonucleoprotein complex composed of three RNA molecules and over 50 proteins. The ribosome is a molecular machine that catalyzes the synthesis of a polypeptide from its substrate, aminoacyl-tRNA. Ribosomes that translate a problematic mRNA, such as that lacking a stop codon, can stall at its 3′ end and produce an incomplete, potentially deleterious protein. *Trans*-translation is known as the highly sophisticated system in bacteria to recycle ribosomes stalled on defective mRNAs and add a short tag-peptide to the C-terminus of the nascent polypeptide as the degradation signal [1–4] (Figure 1). Thus, the tagged polypeptide from truncated mRNA is preferentially degraded by cellular proteases including ClpXP, ClpAP, Lon, FtsH, and Tsp [1, 5–7], and truncated mRNA is released from the stalled ribosomes to be degraded by RNases [8]. The process of *trans*-translation is facilitated by transfer-messenger RNA (tmRNA, also known as 10Sa RNA or SsrA RNA), which is a unique hybrid molecule that functions as both tRNA and mRNA (Figure 2). It comprises two functional domains, the tRNA domain partially mimicking tRNA [9] and the mRNA domain, which includes the coding region for the tag-peptide, surrounded by four pseudoknot structures [10–14]. As predicted from the tRNA-like secondary structure, the 3′ end of tmRNA is aminoacylated by alanyl-tRNA synthetase (AlaRS) like that of canonical tRNA [15, 16]. The function as tRNA is a prerequisite for the function as mRNA, indicating the importance of the elaborate interplay of the two functions [2]. Thus, “*trans*-translation” has been proposed: Ala-tmRNA somehow enters the stalled ribosome, allowing translation to resume by switching the original mRNA to the tag-encoding region on tmRNA. Various questions about the molecular mechanism of this process have been raised. How does tmRNA enter the stalled ribosome in the absence of a codon-anticodon interaction? How is tmRNA switched from the original mRNA in the ribosome? How is the resume codon on tmRNA for the tag-peptide determined? How does tmRNA, 4- or 5-fold larger than tRNA, work in the narrow space in the ribosome? 

Several factors, including EF-Tu [17–20], SmpB [21–23], and ribosomal protein S1 [22–24], have been identified as tmRNA-binding proteins. EF-Tu delivers Ala-tmRNA to the ribosome like aminoacyl-tRNA in translation. Unlike S1 [25–27], SmpB serves as an essential factor for *trans*-translation *in vivo* and *in vitro*. It binds to the tRNA-like domain (TLD) of tmRNA [23, 28–30] and ribosome [21] to perform multiple functions during *trans*-translation, including enhancement of aminoacylation efficiency of tmRNA [22, 23, 31], protection of tmRNA from degradation in the cell [19, 28], and recruitment of tmRNA to the stalled ribosome [21, 23]. NMR studies have revealed that SmpB consists of an antiparallel *β*-barrel core with three helices and flexible C-terminal tail residues that are disordered in solution [32, 33]. 

Here, we review recent progress in our understanding of the molecular mechanism of *trans*-translation facilitated by tmRNA and SmpB, which is being revealed by various chemical approaches such as directed hydroxyl radical probing and chemical modification as well as other biochemical and structural studies.

## 2. *In Vitro Trans*-Translation System

A cell-free *trans*-translation system coupled with poly (U)-dependent polyphenylalanine synthesis was developed using *Escherichia coli* crude cell extracts [2]. Later, several *trans*-translation systems were developed using purified factors from *E. coli* [31, 34, 35] or from *Thermus thermophilus* [25]. These systems have revealed that EF-Tu and SmpB, in addition to the stalled ribosome and Ala-tmRNA, are essential and sufficient for the first few steps of *trans*-translation including the binding of Ala-tmRNA to the ribosome, peptidyl transfer from peptidyl-tRNA to Ala-tmRNA, and decoding of the first codon on tmRNA for the tag peptide. Besides, these systems have also provided a basis for investigating the molecular mechanism of *trans*-translation by chemical approaches.

## 3. Molecular Mimicries of tRNA and mRNA Revealed by Directed Hydroxyl Radical Probing

Ivanova et al. [36] performed chemical probing to analyze the interaction between SmpB and a ribosome. Bases of rRNA are protected from chemical modification with dimethylsulfate or kethoxal by SmpB, indicating that there are two SmpB-binding sites on the ribosome; one is around the P-site of the small ribosomal subunit and the other is under the L7/L12 stalk of the large ribosomal subunit. The capacity of two SmpB molecules to bind to a ribosome is in agreement with results of other biochemical studies [37, 38]. Gutmann et al. [29] showed a crystal structure of *Aquifex aeolicus* SmpB in complex with the tmRNA fragment corresponding to TLD, which confirmed results of earlier biochemical studies showing that TLD is the crucial binding region of SmpB [23]. It also suggested that SmpB orients toward the decoding center of the small ribosomal subunit and that SmpB structurally mimics the anticodon arm. This is in agreement with a cryo-EM map of the accommodated state complex of ribosome/Ala-tmRNA/SmpB [39–41].

**Figure 3:**
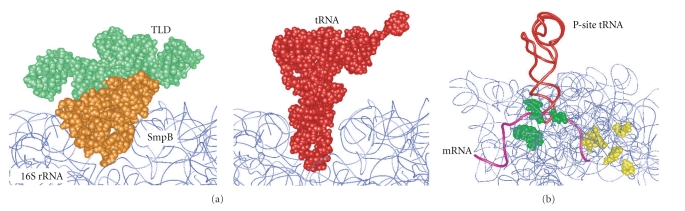
Molecular mimicry of tRNA and mRNA from directed hydroxyl radical probing. (a) Location of TLD-SmpB (left) and tRNA (right) in the ribosomal A-site from directed hydroxyl radical probing [48, 49]. The N-terminal globular domain of SmpB mimics the lower half of tRNA in the A-site. The tertiary structures of TLD-SmpB from *T. thermophilus* [50] and 70S ribosome from *E. coli* [51] were used. (b) Location of the C-terminal tail of SmpB from directed hydroxyl radical probing. Cleavage sites by Fe(II)-tethered A-site and P-site SmpB are colored yellow and green, respectively. The C-terminal tails are located on the mRNA path, suggesting that the C-terminal tail of SmpB mimics mRNA in both the A-site and P-site. P-site SmpB and mRNA are colored red and pink, respectively. The tertiary structure model of 70S ribosome from *T. thermophilus* [52] is used.

A truncation of the unstructured C-terminal tail of SmpB leads to a loss of *trans*-translation activity [42, 43]. In spite of its functional significance, cryo-EM studies have failed to identify the location of the C-terminal tail of SmpB in the ribosome due to poor resolution. We performed directed hydroxyl radical probing with Fe(II)-BABE to study the sites and modes of binding of *E. coli* SmpB to the ribosome (Figure 3). Fe(II)-BABE is a specific modifier of the cysteine residue of a protein, which generates hydroxyl radicals to cleave the RNA chain. Cleavage sites on RNA can be detected by primer extension, allowing mapping of amino acid residues of a binding protein on an RNA-based macromolecule. This is an excellent chemical approach to study the interaction of a protein with the ribosome [44–47]. We prepared SmpB variants each having a single cysteine residue for attaching it to an Fe(II)-BABE probe. Using directed hydroxyl radical probing, we succeeded in identifying the location of not only the structural domain but also the C-terminal tail of SmpB on the ribosome [48]. It was revealed that there are two SmpB-binding sites in a ribosome, which correspond to the lower halves of the A-site and P-site and that the C-terminal tail of A-site SmpB is aligned along the mRNA path towards the downstream tunnel, while that of P-site SmpB is located almost exclusively around the region of the codon-anticodon interaction in the P-site. This suggests that the C-terminal tail of SmpB mimics mRNA in the A-site and P-site and that these binding sites reflect the pre- and posttranslocation steps of *trans*-translation. The probing signals appear at interval 3, residues of the latter half of the C-terminal tail, suggesting an *α* helix structure, which has been predicted from the periodical occurrence of positively charged residues [42]. Consequently, the following model has been proposed. The main body of SmpB mimics the lower half of tRNA, and the C-terminal tail of SmpB mimics mRNA both before and after translocation, while the upper half of tRNA is mimicked by TLD. Upon entrance of tmRNA into the stalled ribosome, the C-terminal tail of SmpB may recognize the vacant A-site free of mRNA to trigger *trans* translation. After peptidyl transfer to Ala-tmRNA occurring essentially in the same manner as that in canonical translation, translocation of peptidyl-Ala-tmRNA/SmpB from the A-site to the P-site may occur. During this event, the extended C-terminal tail folds around the region of the codon-anticodon interaction in the P-site, which drives out mRNA from the P-site.

## 4. Early Stages of *Trans*-Translation

Ala-tmRNA/SmpB forms a complex with EF-Tu and GTP *in vitro*, and this quaternary complex is likely to enter the empty A-site of the stalled ribosome [22]. This complex forms an initial binding complex with the stalled ribosome like the ternary complex of aminoacyl-tRNA, EF-Tu, and GTP does with the translating ribosome. In normal translation, the correct codon-anticodon interaction is recognized by universally conserved 16S rRNA bases, G530, A1492 and A1493, which form the decoding center. When a cognate tRNA binds to the A-site, A1492, and A1493 flip out from the interior of helix 44 of 16S rRNA, and G530 rotates from a syn to an anticonformation to monitor the geometry of the correct codon-anticodon duplex [53]. This induces GTP hydrolysis by EF-Tu, allowing the CCA terminal of tRNA to be accommodated into the peptidyl transferase center. In the context of tRNA mimicry, SmpB should orient toward the decoding center in* trans*-translation. We have recently shown that interaction of the C-terminal tail of SmpB with the mRNA path in the ribosome occurs after hydrolysis of GTP by EF-Tu [49]. According to a chemical probing and NMR study, SmpB interacts with G530, A1492, and A1493 [54]. How these bases recognize SmpB to trigger the following GTP hydrolysis is yet to be studied. It should be noted that recent crystal structures have revealed that these bases recognize the A-site ligands (aminoacyl-tRNAs, IF-1, RF-1, RF-2 and RelE) in different ways during translation [50, 55, 56]. 

Cryo-EM reconstructions of the preaccommodated state of the ribosome/Ala-tmRNA/SmpB/EF-Tu/GDP/ kirromycin complex of *T. thermophilus* have shown that two SmpB molecules present in a complex, one binding to the 50S ribosomal subunit at the GTPase-associated center and the other binding to the 30S subunit near the decoding center [39, 41]. The latter SmpB is not found in the accommodation complex of *T. thermophilus* and *E. coli* [39–41]. Thus, the following model has been proposed: two molecules of SmpB are required for binding of Ala-tmRNA to the stalled ribosome and one of them is released from the ribosome concomitant with the release of EF-Tu after hydrolysis of GTP, so that the 3′-terminal of tmRNA is oriented toward the peptidyl-transferase center. However, several reports have argued against the requirement of two SmpB molecules for *trans*-translation: SmpB has been reported to interact with tmRNA in a 1 : 1 stoichiometry in the cell [57, 58], and crystal structures of SmpB in complex with TLD have been reported to exhibit a 1 : 1 stoichiometry of tmRNA and SmpB [29, 59]. Further studies are required to assess the stoichiometry of SmpB in the preaccommodation state complex.

We have recently shown that the C-terminal tail of SmpB is required for the accommodation of Ala-tmRNA/SmpB into the A-site rather than the initial binding of Ala-tmRNA/SmpB/EF-Tu/GTP to the stalled ribosome [49]. We have also shown that the tryptophan residue at 147 in the middle of the C-terminal tail of *E. coli* SmpB has a crucial role in the step of accommodation. Our results further suggest that the aromatic side chain of Trp147 is required for interaction with rRNA upon accommodation.

It has been shown that *trans*-translation can occur in the middle of an mRNA *in vitro*, although the efficiency of *trans*-translation is dramatically reduced with increase in the length of the 3′ extension from the decoding center [34, 35]. This may be a result of competition of the 3′ extension of mRNA and the C-terminal of A-site SmpB for the mRNA path. The ribosome stalled on the middle of intact mRNA in a cell might be rescued by *trans*-translation via cleavage of mRNA at the A-site [60] or by alternative ribosome rescue systems [61–63].

## 5. Determination of the Resume Codon

In *trans* translation, the ribosome switches template from a problematic mRNA to tmRNA. How does the stalled ribosome select the first codon on tmRNA without an SD-like sequence? It is reasonable to assume that some structural element on tmRNA is responsible for positioning the resume codon in the decoding center just after translocation of peptidyl-Ala-tmRNA/SmpB from the A-site to the P-site. In *E. coli*, the coding region for the tag peptide starts from position 90 of tmRNA, which is 12 nucleotides downstream of PK1. Indeed, PK1 is important for efficiency of *trans*-translation [14], whereas changing the span between PK1 and the resume codon does not affect determination of the initiation point of tag-translation [64]. A genetic selection experiment has revealed strong base preference in the single-stranded region between PK1 and the resume codon, especially −4 and +1 (position 90) [65]. The importance of this region has also been shown by an *in vitro* study [64]. Several point mutations in this region encompassing −6 to −1 decrease the efficiency of tag-translation, while some of them shift the tag-initiation point by −1 or +1 to a considerable extent [59, 60], indicating that the upstream sequence contains not only the enhancer of *trans*-translation but also the determinant for the tag-initiation point. Evidence for interaction between the upstream region and SmpB has been provided by a study using chemical probing [66]. *E. coli *SmpB protects U at position −5 from chemical modification with CMCT. The structural domain of SmpB rather than the C-terminal tail is involved in this protection. The protection at −5 was suppressed by a point mutation in the TLD critical to SmpB binding, suggesting that SmpB serves to bridge two separate domains of tmRNA to determine the resume codon for tag-translation. Mutations that cause −1 and +1 shifts of the start point of tag-translation also shift the site of protection at −5 from chemical modification by −1 and +1, respectively, indicating the significance of the fixed span between the site of interaction on tmRNA with SmpB and the resume point of translation: translation for the tag-peptide starts from the position 5 nucleotides downstream of the site of interaction with SmpB. Such a functional interaction of the upstream region in tmRNA with SmpB is also supported by the results of another genetic study showing that A-to-C mutation at position 86 of *E. coli* tmRNA that inactivates *trans*-translation both *in vitro* and *in vivo* is suppressed by some double or triple mutations in SmpB [67]. In agreement with these studies, recent cryo-EM studies have suggested that the upstream region in tmRNA interacts with SmpB in the resume (posttranslocation) state [68, 69].

The initiation shift of tag-translation can also be induced by the addition of a 4,5- or 4,6-disubstituted class of aminoglycoside such as paromomycin or neomycin [70, 71], which usually causes miscoding of translation by binding to the decoding center on helix 44 of the small subunit to induce a conformational change in its surroundings [72]. Aminoglycosides also bind at helix 69 of the large subunit, which forms the B2a bridge with helix 44 in close proximity of the decoding center in the small subunit, to inhibit translocation and ribosome recycling by restricting the helical dynamics of helix 69 [73]. Taken together, these findings suggest the significance of interaction of the proximity of the decoding center with any portion of SmpB or tmRNA for precise tag-translation. It should be noted that hygromycin B, which binds only to helix 44, does not induce initiation shift of tag-translation [71].

## 6. Trajectories of tmRNA/SmpB

Along with the functional mimicry of TLD/SmpB, a similar behavior of tmRNA/SmpB to that of canonical tRNA+mRNA in the ribosome through several hybrid states, A/T, A/A, A/P, P/P, and P/E, has been assumed. Cryo-EM studies have shown the location of the complex of tmRNA with the main body of SmpB in the A/T and A/A states [39, 40], and a directed hydroxyl radical probing has revealed the positions of SmpB in the A/A and P/P states [48]. The existence of stable SmpB binding sites in the A-site and P-site suggests the requirement of translocation, as in canonical translation. It might possibly involve EF-G. Concomitantly with translocation, mRNA and P-site tRNA are released from the stalled ribosome [74]. Considering the different C-terminal tail structures of A-site SmpB and P-site SmpB, the C-terminal tail would somehow undergo conformational change from the extended form to the folded form [48].

The next translocation is thought to move tmRNA/SmpB to the E-site. These ribosomal processes should involve extensive changes in the conformation of tmRNA [75] as well as in the modes of interactions of tmRNA with SmpB and the ribosome [76, 77]. According to chemical probing studies, secondary structure elements of tmRNA remain intact in a few steps of *trans*-translation including pre- and posttranslocation states [77–79]. Another study has suggested 1 : 1 stoichiometry of tmRNA to SmpB throughout the processes of translation for the tag peptide [80]. Recently, the movement of tRNA during translocation has been revealed by using time-resolved cryo-EM [81]. Not only classic and hybrid states but also various novel intermediate states of tRNAs were revealed. Although the intermediate states during *trans*-translation remain unclear, results of future structural studies including chemical approaches should reveal tmRNA/SmpB and ribosome dynamics.

## 7. Conclusion

**Figure 4:**
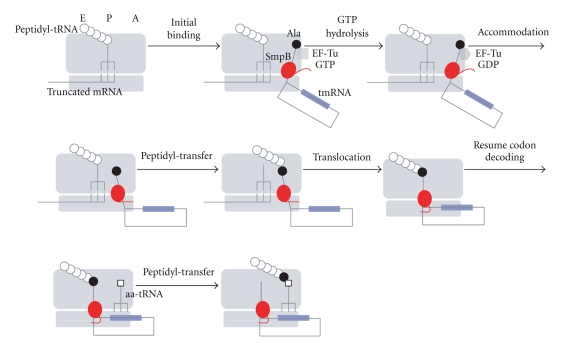
A model of the early stages of *trans*-translation. The C-terminal tail of SmpB is not located on the mRNA path in the processes before accommodation. After GTP hydrolysis by EF-Tu, the C-terminal tail is located on the mRNA path mimicking mRNA to recognize the stalled ribosome free of mRNA. Following translocation of tmRNA/SmpB from the A-site to P-site, the C-terminal tail undergoes drastic conformational change to accommodate the resume codon of tmRNA into the decoding center. SmpB and the tag-encoding region are shown by red and blue, respectively. White circles indicate amino acids encoded by truncated mRNA, and a white square indicates amino acid designated by the resume codon of tmRNA.

Various chemical approaches in addition to cryo-EM and X-ray crystallographic studies have been revealing the molecular mechanism of *trans*-translation. tmRNA forms a ribonucleoprotein complex with SmpB, which plays an essential role in *trans*-translation. Based on a directed hydroxyl radical probing towards SmpB, we have proposed a novel molecular mechanism of *trans*-translation (Figure 4). In this model, an elegant collaboration of a hybrid RNA molecule of tRNA and mRNA and a protein mimicking a set of tRNA and mRNA facilitates *trans*-translation. Initially, a quaternary complex of Ala-tmRNA, SmpB, EF-Tu, and GTP may enter the vacant A-site of the stalled ribosome to trigger *trans*-translation, when a set of Ala-TLD of tmRNA and the main body of SmpB mimicking the upper and lower halves of aminoacyl-tRNA, respectively, recognizes the A-site free of tRNA. After hydrolysis of GTP by EF-Tu, the C-terminal tail of SmpB mimicking mRNA interacts with the decoding center and the downstream mRNA path free of mRNA, allowing Ala-TLD/SmpB to be accommodated. While several proteins including SmpB have been proposed to mimic tRNA or its portion, SmpB is the first protein that has been shown to mimic mRNA. SmpB is also the first protein of which stepwise movements in the ribosome are assumed to mimic those of tRNA in the translating ribosome.

Our model depicts an outline of the *trans*-translation processes in the ribosome, although the following issues should be addressed. How do the intermolecular interactions between tmRNA and ribosome, between tmRNA and SmpB, and between ribosome and SmpB as well as the intramolecular interactions within tmRNA and within SmpB change during the course of the *trans*-translation processes? Is EF-G required for translocation of tmRNA/SmpB having neither an anticodon nor the corresponding codon from the A-site to the P-site? If EF-G is required, how does it promote translocation? These questions remain to be answered in the future works.

## Abbreviations

AlaRS: Alanyl-tRNA synthetase
BABE: 1-(*p*-bromoacetamidobenzyl)- ethylenediaminetetraacetic acid
CMCT: 1-cyclohexyl-3-(2-morpholinoethyl) carbodiimide metho-*p*-toluene sulfonate
cryo-EM: Cryo-electron microscopy
EF-Tu: Elongation factor Tu
EF-G: Elongation factor G
PK: Pseudoknot
TLD: tRNA-like domain.
